# SeedlingNet: A colour-based segmentation approach towards classification of plant species seedlings

**DOI:** 10.1016/j.mex.2026.103883

**Published:** 2026-03-24

**Authors:** Pushpa B R, Bhavya K R, Manohar N

**Affiliations:** a Department of Computer Science, Amrita School of Computing, Amrita Vishwa Vidyapeetham, Mysuru 570026, India; b Department of Computer Science and Engineering, GSCSE, GITAM University, Bengaluru, India

**Keywords:** Deep learning, Plant seedlings, Color models, Thresholding, Classification

## Abstract

Early identification of plant species in uncontrolled conditions can be complex due to the complex background and morphological diversity of a plant's leaves. Agriculture plays an important role in our daily life to produce food, develop economies, international trade, research, and promote global sustainability. In this work, classification of plant species is performed based on a proposed segmentation method using a colour thresholding technique to separate the plant regions in images captured in low lighting and a complex background. The models include DenseNet121, NasNet Mobile, MobileNet, RegNet, and XceptionNet, trained on the proposed segmentation method and raw image samples, and compared for their effectiveness. The dataset, comprising 4750 images of 12 different types of plants, their seedlings, and weeds, is sourced from Kaggle. The results demonstrate that the model's classification performance using the proposed segmentation method consistently outperformed with the non-segmented images, confirming the need for segmentation to improve model accuracy.

## Highlights of the proposed method

• Proposed a segmentation method for extracting plant parts from complex environments with low lighting conditions.
• Compared the proposed segmentation model against other existing segmentation methods.
• Different deep learning models are trained on segmented and non-segmented images for classifying various seedlings to accurately determine an effective transfer learning model with the best possible recognition rate.

## Graphical abstract

**Figure:**
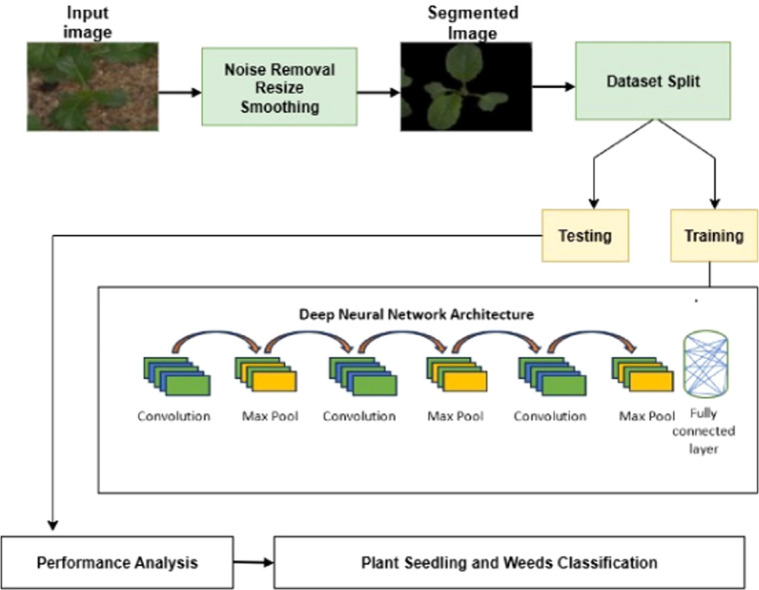

**Specifications table**

**Table:** 

Subject area	Computer Science
More specific subject area	Computer Vision, Image Segmentation, Deep Learning in Agriculture
Name of your method	SeedlingNet
Name and reference of the original method	Color thresholding and morphological operators
Resource availability	Dataset: Plant Seedlings Dataset (Kaggle)https://www.kaggle.com/c/plant-seedlings-classification

## Background

[1] Computer vision technology is one of the key components of agricultural productivity with respect to cost efficiency through the identification of weeds, predicting plant maturation, identifying diseases, and classifying crops. The agricultural sector has provided 17.6% to 20.2% gross domestic product (GDP) to the country over the last 3 years [23]. Therefore, distinguishing between types of crops, separating crops from weeds, identifying crops contributing to the Global economy, and providing better functionality within the industry are essential for food quality and crop productivity. The presence of weeds competing with crops for water, nutrients, and sunlight impacts crop yields, making weed control a critical factor in crop production. The farmers, having limited or no knowledge of crops, experience decreased production when trying to distinguish between crops and weeds during the seedling stages of growth. Therefore, utilizing computer-based technologies to overcome these barriers that farmers face in the agricultural industry is necessary for success. Conventional computer vision methods are manually crafted features that are complex and time-consuming. On the other hand, deep learning models automatically learn features from the images, reducing human intervention and potentially improving performance. In addition, deep learning models can handle large datasets with images posing different challenges [14]. Adopting these technologies would significantly enhance agricultural productivity, with estimates suggesting that every 1% increase in production could reduce poverty by 0.6–1.2% [1]. In [2], three pre-trained deep learning models were utilised to perform plant seedling classification on the benchmark datasets. The homogenous and heterogeneous deep learning models are fused for the plant species classification in real-time environments [3]. The study [4] shows the effectiveness of modified deep learning models for classifying native plant seedlings from the Philippines. In [5], an Extreme Learning Machine (ELM) was adapted to classify plant diseases using machine learning methods based on texture feature extraction. The experiments were carried out on PlantVillage datasets of tomato plants [6] developed an automated system using color and textural features to classify diseased, healthy potato and grape leaves [7] Different machine learning models are investigated for the early detection and classification of maize leaf diseases [[8], [9]]. The EfficientNetB0 model was used to classify crops and weed seedlings for early-stage weed management in agriculture. A [10] CNN model trained on a farm field to identify and classify crops and weeds demonstrates the potential of deep learning models. The work [11] explored the R-CNN model for detecting conifer seedlings in forest areas using drone imagery. Different pre-trained models have been tested on the benchmark datasets, including LeafSnap, Malayakew, Flavia, Folio, Swedish, Plant Village datasets, and Plantclef captured in a constrained environment. However, they do not address challenges such as scale variation, viewpoint or orientation, cluttered background, different lighting conditions, compound leaf structures, or whole plant images. Most of the works in the literature focused on deep learning models for plant species classification, disease detection, and recognizing nutrient deficiency, etc., without performing enhancement. In some works, existing segmentation methods are utilised, which can be subjective, require human intervention, be inaccurate, and time-consuming [[16], [19], [27], [28]].

## Method details

This segmentation process involves a combination of thresholding, using the HSV color space, and morphological operations that allow the separation of plants from complex backgrounds. The first step consists of changing the original RGB image to the HSV image. This will improve contrast between the image of the leaf and the other components, and allow for a greater possibility of separating pixel areas of characteristic colour and range of intensity which correspond to green leaves. After converting to HSV, the H, S, and V channels (hue, saturation, and value) can each be thresholded individually. This will produce a binary mask for each channel that represents pixel areas that meet the conditions of being healthy green in colour and having an acceptable range of intensity. After creating binary masks for each of the three channels, they can be combined using a logical AND operator to create a binary mask that represents only the pixels that meet the conditions for all three channels. This composite binary mask must then be cleaned up through the use of morphological operations, such as erosion and dilation. Erosion will eliminate small noise in the binary mask, while dilation will help to fill in small gaps in the binary mask, providing a smoother contour around the leaf. Once cleaned up using morphological operations, the accurate tracing of the outline of the leaf through contour detection will provide the necessary information to create a clean final segmented leaf in the original image. This entire workflow can be employed to accurately segment leaves from images containing complex backgrounds or where the lighting conditions are uneven. Fig. 2 presents the workflow of the proposed segmentation method.

## Dataset description

**Fig. 1:**
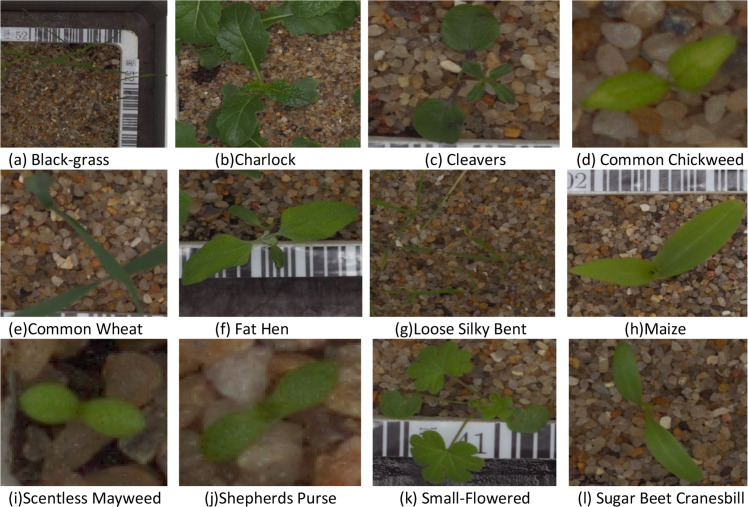
Sample images of the plant seedlings dataset.

The dataset used for the proposed work is sourced from the Kaggle repository titled "Plant Seedlings Classification", a widely used benchmark for plant species identification tasks in agricultural computer vision research. The dataset comprises 4750 high-resolution RGB images, encompassing twelve species of common plants and weeds found in agricultural fields. The images capture seedlings at various early growth stages, providing a diverse representation of plant morphology from the cotyledon stage to early true leaf phases. The dataset has images labeled to indicate the plant species and an approximate spatial resolution of ten pixels per millimeter, making it suitable for visual and quantitative analyses of traits. The images were taken under semi-controlled conditions in the laboratory at the Falkenberg Agroecology Research Facility, where seedlings are grown in pots containing soil mixed with small stones and organic matter. Image acquisition took place over more than twenty days, so that multiple development stages and growth patterns through time for each species could be represented. However, the dataset presents many visual and computational challenges, including low lighting levels, different soil types as backgrounds, growth stages, and a high level of similarity between plant species, as well as high variability within classes. Other issues are motion blur and partial obstructions of the plant by either leaf overlap or soil particles surrounding the plant. Additionally, there is a significant similarity in color tones between the background and leaf foliage of the samplings used, particularly concerning soil impurities that are green in color. These conditions indicate that thresholding or edge segmentation alone will not be a viable technique for separating background from a leaf until preprocessing has been performed on the images. A more sophisticated segmentation method is needed to successfully isolate the areas of the plant for analysis. In addition, the details about the species used in the dataset are shown in Table 1, and examples of images of these species can be found in Fig. 1.

**Table 1:** Details of plant species available in the dataset.

Sl.no	Name	Sample Size
1	Black-grass	263
2	Charlock	390
3	Cleavers	287
4	Common Chickweed	611
5	Common Wheat	221
6	Fat Hen	475
7	Loose Silky Bent	654
8	Maize	221
9	Scentless Mayweed	516
10	Shepherds Purse	231
11	Small-Flowered	496
12	Sugar Beet Cranesbill	385

**Fig. 2:**
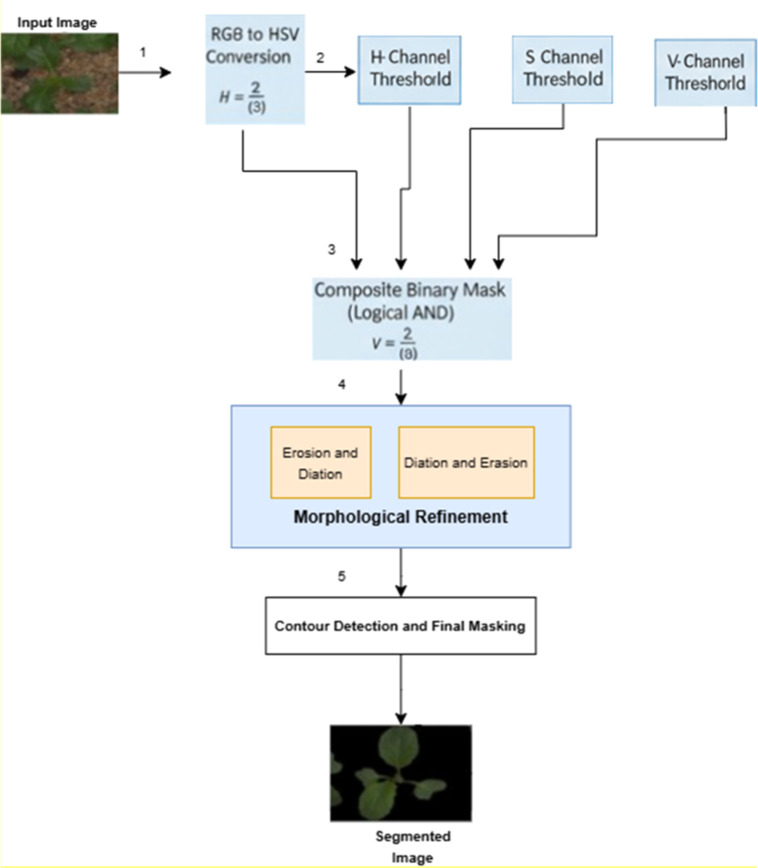
Proposed seedlingnet model.

## SeedlingNet – segmentation method

RGB to HSV Colour Space Transformation: Initially, the input RGB colour image is converted to HSV colour space [30], which simplifies the analysis of colour-specific features and filters out irrelevant details caused by illumination variations. Let the input image be represented as IRGB(x,y), where (x,y) denotes the spatial pixel coordinates in the RGB color space. The conversion from RGB to HSV color space is denoted in eq (1):

(1) $$I_{RGB}\left(x,y\right)=\left[R\left(x,y\right),G\left(x,y\right),B\left(x,y\right)\right]$$

where R(x,y),G(x,y),B(x,y) denote the red, green, and blue channel intensities at pixel location (x,y) .The transformation to HSV is given by:

(2) $$I_{HSV}\left(x,y\right)=\left[H\left(x,y\right),S\left(x,y\right),V\left(x,y\right)\right]=RGBtoHSV(I_{RGB}\left(x,y\right)$$

In this space, hue ${}^{\prime }H^{\prime }$ represents the dominant wavelength, saturation ${}^{\prime }S^{\prime }$ captures color purity, and value ${}^{\prime }V^{\prime }$ encodes brightness. This separation allows for more targeted filtering of vegetation regardless of illumination differences.

Threshold-Based Masking: In order to accurately separate and identify the plant regions from the other pixel values, threshold values are established empirically for the H, S, and V channels through channel-wise binary masking. The purpose of creating thresholds is to define the hue and saturation ranges of green plants while eliminating the hues and saturation ranges associated with the non-plant areas, such as soil, shadows, and background clutter. Thresholding not only increases the contrast between the vegetation and its surroundings but also creates an initial binary mask, which will be further processed for segmentation in the pipeline. The color masks derived from the threshold values can be seen in Eqs. (3), (4), and (5).

(3) $$M_{H}\left(x,y\right)=\left\{ \begin{array}{c} 1\;if\;H_{min}\leq H\left(x,y\right)\leq H_{max}\\ \;0\;otherwise\; \end{array}\right.$$

(4) $$M_{S}\left(x,y\right)=\left\{ \begin{array}{c} 1\;if\;S_{min}\leq S\left(x,y\right)\leq S_{max}\\ \;0\;otherwise\; \end{array}\right.$$

(5) $$M_{V}\left(x,y\right)=\left\{ \begin{array}{c} 1\;if\;V_{min}\leq V\left(x,y\right)\leq V_{max}\\ \;0\;otherwise\; \end{array}\right.$$

These masks respectively filter hue values corresponding to green tones, remove desaturated regions, and eliminate pixels that are excessively dark or overexposed. A final composite mask is then formed via logical AND operation as expressed in eq. (6).

(6) $$M\left(x,y\right)=M_{H}\left(x,y\right)\wedge M_{S}\left(x,y\right)\wedge M_{V}\left(x,y\right)$$

Foreground Isolation: The binary mask M(x,y) is applied to the original RGB image to isolate plant regions. This operation preserves only the plant pixels, while suppressing the background by replacing it with black, and produces a visually interpretable segmented image as presented in eq. (7).

(7) $$I_{segmented}\left(x,y\right)=\left\{ \begin{array}{c} I_{RGB}\left(x,y\right),\;if\;M\left(x,y\right)=1\\ \left[0,0,0\right],\;otherwise \end{array}\right.$$

Morphological Refinement: Despite the HSV-based masking, residual noise and artifacts may persist due to illumination inconsistencies or background interference. To address this, morphological operations are employed to refine the binary mask using a 5 × 5 structuring element (SE). The mask is processed through an opening operation followed by a closing operation, mathematically defined in (8), (9).

(8) $$M_{open}\left(x,y\right)=\left(M⊖\text{SE}\right)⊕\text{SE}$$

(9) $$M_{clean}\left(x,y\right)=\left(M_{open}⊖\text{SE}\right)⊕\text{SE}$$

where, (M⊖SE)⊕SE denotes erosion and dilation, respectively. This process removes specks and fills holes, yielding a cleaner and more contiguous plant region.

Contour Detection and Final Masking: Contour analysis is conducted using cv2.findContours() to detect the boundaries of connected white regions in $M_{clean}\left(x,y\right)$ which are likely to represent leaves or seedlings. The detected contours are drawn and filled on a blank canvas to produce a final refined mask. This refined mask is then applied in a second masking operation as given in eq. (10).

(10) $$I_{final}\left(x,y\right)=\left\{ \begin{array}{c} I_{segmented}\left(x,y\right),\;if\;M_{clean}\left(x,y\right)=1\\ \left[0,0,0\right],\;otherwise \end{array}\right.$$

**Fig. 3:**
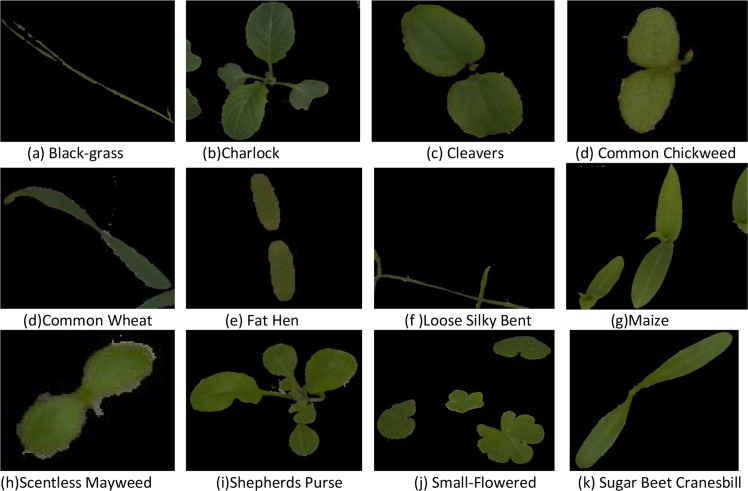
Segmentation results of the plant seedlings and weeds.

The outcome is a segmented image that retains only the validated, connected plant regions, while eliminating background clutter and noise. This segmentation pipeline provides a robust basis for further analysis, including classification and morphological feature extraction, and demonstrates high reliability in varying outdoor lighting and environmental conditions. Fig. 3 represents the segmentation results obtained from the proposed method.

## Method validation

**Fig. 4:**
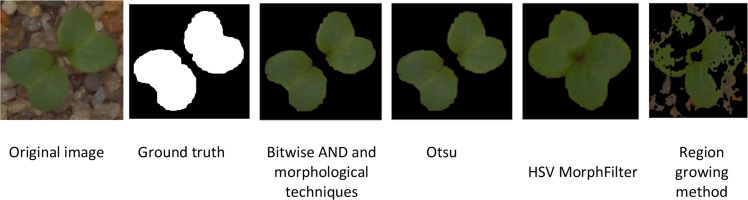
Segmentation results of the existing methods.

The proposed segmentation approach was validated on a plant seedlings dataset to test the method's reliability and robustness by simulating experiments in environments with the potential to influence the outcome of the results. The results have been compared to the ground truth for each image, providing an unbiased, accurate assessment of the performance of the proposed method. The segmentations were also compared against four different existing segmentation methods, including the combination of bitwise and morphological techniques, Otsu thresholding, morphological techniques combined with HSV colour thresholding, and region growing segmentation and region growing methods as shown in Fig. 4. The segmentation techniques were selected for comparison because they are commonly used in the field of plant image processing and have widespread use of these techniques in existing literature. The combination of bitwise AND with morphological operations is a straightforward method of segmenting plant leaf areas, where the initial mask is generated based on RGB/intensity information and further refined through the use of erosive/dilative/opening/closing morphological operations to reduce noise and enhance edge smoothness. Otsu thresholding relies solely on intensity histogram analysis to determine the best single global threshold value between foreground and background. Therefore, it only works well under uniform lighting conditions. The HSV colour threshold provides a better method of segmenting the green leaf pixels because it works in the colour space where hue is independent of brightness, so this permits more successful extraction of green leaf pixels. Morphological filtering cleans the mask obtained from HSV and enables clean segmentation of green leaf pixels using adjacent seed pixels as a basis. Finally, region growing works by starting with seed pixels that were placed inside leaf areas. As additional neighbouring pixels are added to the seeds based on object pixels having colours/intensities that are similar to the seed pixels, the natural boundaries of each leaf will be maintained if the pixels have relatively uniform colours/intensities.

To further evaluate this method and compare it quantitatively against other methods, different metrics were used, including: Dice Coefficient, Precision, Recall, Accuracy, and Intersection over Union (IoU). In combination, each metric measures how well predicted masks overlap with the ground truth, as well as how accurately they are aligned to each other with respect to boundary alignment and their ability to provide the most accurate results based on identifying plants, while avoiding false-positive and false-negative identification. The Dice Coefficient and IoU are metrics primarily used for evaluating general segmentation performance based on determining how many pixels overlap spatially between the predicted mask and the true mask. The precision and recall provide a more detailed representation of the number of correctly identified pixels and how many pixels were missed or incorrectly identified. The accuracy is an overall measure of the percentage of pixels that have been correctly classified across the entire size of the image. Table 2 indicates that the proposed method achieved the highest segmentation performance compared to the existing models with a highest accuracy of 96.2%, dice of 95.02%, and IoU of 92.52%. The combination of bitwise ‘AND’ & the morphological operations to create a segmented image produced good results after experimenting with various kernel sizes, with the most successful kernel size being 3 × 3; it effectively reduced background noise while at the same time preserving the green regions of plant species. The HSV color thresholding with morphological filtering, particularly with hue ranges between 25 and 85, produced the most stable and consistent results across diverse lighting conditions. Morphological closing enhanced region continuity, resulting in smoother plant boundaries and reduced fragmentation. The region growing method was highly sensitive to seed point selection, performing well for uniform leaf textures and minimal background complexity; its reliability dropped in cluttered or noisy scenes. Otsu thresholding showed low results compared to other methods, but by combining with color space pre-filtering, accuracy declined significantly in shadowed or low-light regions, highlighting its sensitivity to global intensity variations, making it less robust for outdoor plant images.

**Table 2:** Comparison of segmentation accuracy across existing and the proposed method.

Method	Dice	IoU	Accuracy	Precision	Recall
Bitwise and morphological techniques	91.71	84.70	92.36	91.94	84.74
Otsu method	60.71	62.5	58.18	64.94	61.74
HSV MorphFilter	88.20	89.04	86.36	85.11	86.76
Region growing	76.89	62.46	63.14	79.55	62.64
**SeedlingNet**	**95.02**	**92.52**	**96.2**	**97.36**	**95.24**

## Deep learning-based classification

Upon implementing plant segmentation, we assessed the classification performance of various deep learning models and compared their accuracies. The models assessed were: DenseNet121, NasNet Mobile, MobileNetV2, RegNet, and XceptionNet. DenseNet121 consists of 121 layers and has been adjusted to the input image size and has a dense layer containing 1024 neuron, with a dropout rate of 0.5, then has a 10 neuron layer which is SoftMax activated; furthermore, it has three dense blocks connected by transition layers and one global average pooling layer to reduce the spatial dimensions of the pooling layer; DenseNet121 has been trained using Adam optimiser [[12], [24]]. The NasNet Mobile model was developed using a Neural architecture search (NAS) for efficient mobile application and also has the modular reduction layer, which supports several repeated elements [[13], [25]]; and therefore, the NasNet Mobile also supports skip connections, use of the bottleneck layer and reduced filter sizes to enable processing images of different sizes dependent upon the image processing constraints, for simplification MobileNetV2 uses inverted residual blocks in a linear bottleneck manner and depth-wise separable convolutions to provide a lightweight architecture for both maximum accuracy and acceptable accuracy, furthermore this model has also been trained using categorical-cross-entropy loss function and Adam optimiser [26].

## Dataset partition and parameters optimization

In this study, we compiled a dataset of 4750 high-resolution images of plants, which included 12 types of seedlings and weeds. This dataset was separated into three distinct parts so that all models could be trained using balanced samples while also being able to accurately assess how well they generalize. The amount of the dataset aside for training was equal to 80%, and 10% divided equally between validation and test data. Both training and testing were performed in a Google Colab environment, using the enhanced computational power of GPUs to speed up the experiments. Hyperparameters included optimised learning rates, batch sizes, selection of optimisers, and regularisation methods to employ while training. A batch size of 32, a learning rate of 0.001 and 25 training epochs were used for all of the experiments. The study utilised transfer learning methodologies to train pre-trained Convolutional Neural Networks (CNNs) such as XceptionNet, MobileNetV2 [17], NASNet-Mobile, DenseNet121 [15], and RegNet. For each model, the last classification layer was removed and replaced with the appropriate number of classes corresponding to the classification categories in the Plant Seedling dataset. Additionally, when fine-tuning, the weights of the newly added classification layers were modified while the pre-trained layers' weights remained either frozen or partially trainable, depending upon the Model’s efficacy as measured on the validation data. This methodology ensures that low-level visual features pre-trained on very diverse data sets would be preserved while allowing the models to be adapted towards specific tasks. Training of the models was accomplished through back propagation, with optimisers selected to optimise classification loss through minimisation.

Table 3 represents the total number of parameters represented as well as the amount of time taken to train each deep learning model for both raw and segmentation images. It provides a useful overview of how deep learning models compare in terms of performance, as it relates to time and parameter count. The Densenet121 deep learning model results the highest amount of time spent training when using both raw and segmented images—14,332 s and 18,110 s, respectively. In terms of parameter counts, Densenet121 also had the highest number of all models represented, with a total of 80,99,404 parameters [[20], [21]]. The NasNet Mobile and MobileNet deep learning models were comparatively quite similar in terms of total training time needed when trained using raw images. The only difference was that the NasNet Mobile model required 14,025 s to complete its training, while the MobileNet model completed its training in 5206 s. When segmented images are evaluated, the MobileNet model again had an increased level of efficiency in the number of seconds it took for the training process (8654 s versus 15,195 s), thus demonstrating that MobileNet's computational advantage over both the NasNet Mobile and Densenet121[22]. The total number of parameters for the MobileNet model was also lower than that of NasNet Mobile—MobileNet with 3582,028 and NasNet Mobile with 5364,384. It takes RegNet longer to process raw images than MobileNet and XceptionNet, with times of 14,865 s vs 5206 s and 1956 s, respectively; however, when using segmented images, RegNet takes less time than DenseNet121 (18,110 s) to complete segmentation (i.e., 13,978 s). RegNet has the highest number of parameters of all the models studied (31,511,116 parameters). For both segmented and non-segmented images, the fastest processing model is XceptionNet, taking 1956 s for raw images and 2668 s for segmented images; furthermore, XceptionNet has a lower number of parameters than DenseNet121, NasNet, and RegNet (4499,524).

**Table 3:** Deep learning models-training time and number of parameters.

Model	*Training time- Raw images in sec*	*Training time- Segmented images in sec*	Number of parameters
Densenet121	14,332	18,110	8099,404
NasNet mobile	14,025	15,195	5364,384
MobileNet	5206	8654	3582,028
RegNet	14,865	13,978	31,511,116
XceptionNet	1956	2668	4499,524

## Performance metrics

It provides a thorough analysis of the classification algorithm's performance. It compares the actual labels of the data with the predictions a model made on a set of test data [[18], [29]]. Accuracy is calculated by evaluating various parameters, including true positive (TP), true negative (TN), false positive (FP), and false negative (FN), as in eq (11).

(11) $$Accuracy=\frac{\text{TP}+\text{TN}\;}{\text{TP}+\text{TN}\;+\text{FP}+\text{FN}}$$

TP refers to accurate data labels that were accurately anticipated in relation to the ground truth. FP are negative data labels that were incorrectly predicted and assigned to an incorrect image label category. The negative data samples with accurate predictions are called TN. FN denotes the positive data labels that were incorrectly predicted. The accuracy of various deep learning models is depicted in Table 4. All models were trained with a 32-batch size and for 25 epochs. The RegNet model has the lowest accuracy of 68.43% for raw images and 77.64% for segmented images compared to other models. The NasNet mobile and MobileNet models have similar accuracy for raw and segmented images, with MobileNet having a slightly higher accuracy of 93.72% for raw images and 97.16% for segmented images. Among the models tested, the XceptionNet model has the highest accuracy, 96.08% for raw photos, but a slightly lower accuracy for segmented images.

**Table 4:** Performance accuracy of various deep learning models.

Models	Accuracy -Raw images	Accuracy- Segmented images
Densenet121	83.49%	93.14%
NasNet Mobile	92.23%	96.45%
MobileNetV2	93.72%	97.16%
RegNet	68.43%	77.64%
XceptionNet	**96.08%**	**94.13%**

**Fig. 5:**
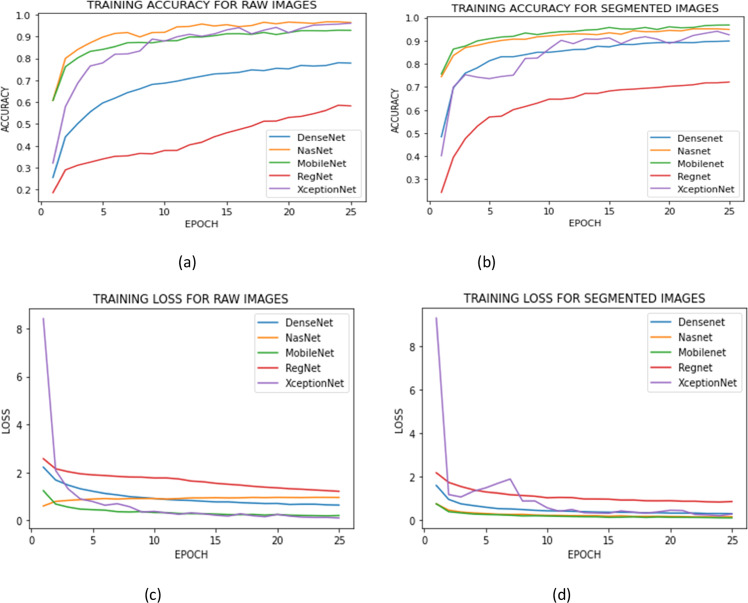
(a)training accuracy of raw images (b) training accuracy for segmented images (c) training loss of raw images (b) training loss for segmented images.

Further, the accuracy of training and validation sets and loss of training and validation set of various deep learning models with and without segmented images are shown in Fig. 5. Fig. 5(a) represents the training accuracies for the raw images. In the first epoch, the Densenet model's accuracy is 25.5%; it then steadily increases over the next epochs, reaching a peak accuracy of 83.49% in the 25th epoch. The NasNet Mobile model, on the other hand, starts with a relatively high accuracy of 60.70% in the first epoch and continues to improve over the training period. It achieves the highest 92.23% in the 25th epoch. The Mobilenet model starts with an accuracy of 60.76% in the first epoch and shows significant improvement up to the 7th epoch. After that, it maintains a stable accuracy between 90% and 93% until the 25th epoch. The Regnet model starts with an accuracy of 18.63% in the first epoch and shows gradual improvement over the subsequent epochs. It achieves a peak accuracy of 58.56% in the 25th epoch. Finally, the Xceptionnet model shows an initial accuracy of 32.13% in the first epoch, which improves significantly in the following few epochs and leads to fluctuating for a certain number of epoches and finally stands with a 96.08 accuracy in the 25th epoch.

The training losses of the raw images are shown in Fig. 5(c). At the end of the 25th epoch, Densenet's training loss has steadily dropped from an initial value of 2.2258 to 0.6453. NasNet experienced varying losses in the first eleven epochs, but by the conclusion of the 25th epoch, the loss had consistently dropped to 0.9640. During the first few epochs, Mobilenet's loss decreased quickly before stabilising with variations around 0.2. Regnet's loss decreased over time, however it did so less steadily than Densenet's and NasNet's, varying in the later epochs about 1.8. Xceptionnet started off with a significant loss but reduced quickly over the course of the first few epochs, eventually decreasing to 0.1076 by the end of the 25th epoch.

The Fig. 5(b) represents the training accuracies of segmented images. DenseNet121 has the lowest accuracy initially, with an accuracy of 48.39%. Still, it gradually improves over time, and in epoch 25, it has the fourth highest accuracy of all the models, with an accuracy of 89.89%. NasNet mobile has a relatively high initial accuracy of 74.44% in the initial epochs, and it gradually improves over some epoches, with an accuracy of 95.17% in epochs 23 and 24 before dropping slightly to 94.92% in epoch 25. MobileNet starts with an accuracy of 75.63% in the first epoch, and its accuracy improves significantly in the first few epochs, reaching 95.77% in epoch 15. It then fluctuates slightly but eventually settles at an accuracy of 96.85% in epoch 25, which is the highest accuracy among all the models. RegNet has the lowest accuracy of all the models throughout the 25 epochs, with an accuracy of only 24.33% in the first epoch. However, it gradually improves over time and reaches an accuracy of 72.16% in epoch 25. XceptionNet has an accuracy of 40.22% in the initial epoch, but it improves significantly in the first few epochs, reaching an accuracy of 90.20% in epoch 10. It then fluctuates but eventually settles at an accuracy of 92.58% in epoch 25.

## Limitations

The deep learning models were evaluated on seedlings and weeds at the plants early growth stages, which may limit their applicability to later or more variable growth conditions.

## Declaration of competing interest

The authors declare that they have no known competing financial interests or personal relationships that could have appeared to influence the work reported in this paper.

## Data Availability

Dataset utilised for the proposed work are collected from the public repository-"Mendeley"
